# Books and Magazines

**Published:** 1898-08

**Authors:** 


### Books and Magazines.

RECENT novels of American life form the subject of an entertaining and on the whole discriminating paper in the *Edinburgh Review*, which American readers will find in the *Living Age* for July 16.

Sir Henry Irving's lecture on The Theatre in Its Relation to the State, delivered at the University of Cambridge June 15, is reproduced in full in the *Living Age* for July 30. No one could be more competent than the distinguished actor to treat such a subject.

---

THE *American Monthly Review of Reviews* for August reviews the Santiago campaign by land and by sea from start to finish. Winston Churchill, who wrote so acceptably on Admiral Dewey for the June *Review*, describes in this number the wonderful battle with Cervera's fleet, and his article is illustrated in part from Hemment's remarkable photographs of the Spanish ships taken the day after the fight. John A. Church, formerly of the *Army and Navy Journal*, contributes a full account of the Santiago land fighting, and his article also is illustrated from new photographs. Park Benjamin writes on the work cut out for the Eastern squadron under Commodore Watson. Altogether, the *Review* again shows its ability to keep well abreast of all important military and naval movements, and to exhibit a clean pair of heels to all its competitors in magazinedom.

The cartoons reproduced in this number from Spanish journals serve to indicate the density of popular ignorance in Spain as to the facts of the present war. For instance, one cartoon shows Cervera's fleet as successfully slipping past Sampson at Santiago; another represents Cervera as having Schley bottled up; while in a third Admiral Dewey figures as a rat caught in Spain's Philippine trap.

